# Mesoporous Pt nanospheres with designed pore surface as highly active electrocatalyst†
†Electronic supplementary information (ESI) available. See DOI: 10.1039/c5sc03779d



**DOI:** 10.1039/c5sc03779d

**Published:** 2015-12-08

**Authors:** Bo Jiang, Cuiling Li, Victor Malgras, Masataka Imura, Satoshi Tominaka, Yusuke Yamauchi

**Affiliations:** a World Premier International (WPI) Research Center for Materials Nanoarchitectonics (MANA) , National Institute for Materials Science (NIMS) , 1-1 Namiki , Tsukuba , Ibaraki 305-0044 , Japan . Email: Yamauchi.Yusuke@nims.go.jp; b Faculty of Science and Engineering , Waseda University , 3-4-1 Okubo, Shinjuku , Tokyo 169-8555 , Japan

## Abstract

A novel strategy for large-scale synthesis of shape- and size-controlled mesoporous Pt nanospheres (MPNs) through a slow reduction reaction in the presence of surfactant is reported here for the first time. The slow reduction reaction exclusively results in well-defined mesoporous architectures distinctly different from the dendritic constructions reported previously. More importantly, abundant catalytically active sites are created on the highly accessible mesoporous surfaces by the selective adsorption of bromide ions. The MPNs prepared by using the new synthetic route not only show superior electrochemical performance toward methanol oxidation reaction and oxygen reduction reaction, but also exhibit extremely high structural thermostability, which makes them promising catalysts for industrial applications.

## Abstract 2

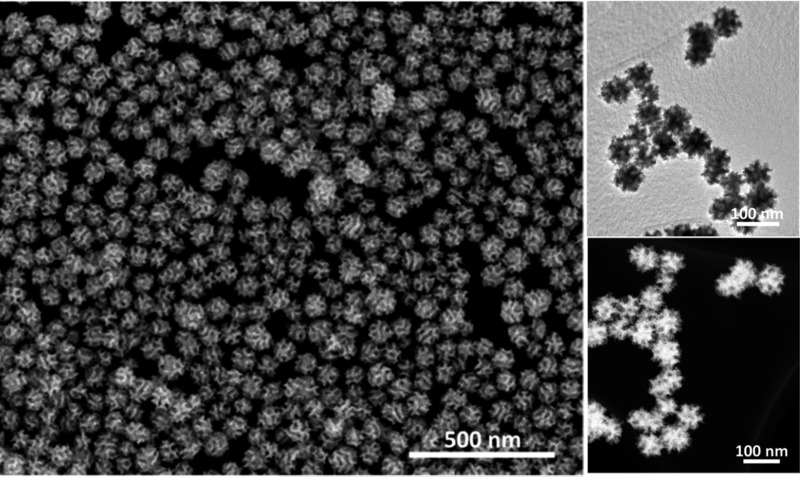
A novel strategy for large-scale synthesis of shape- and size-controlled mesoporous Pt nanospheres (MPNs) through a slow reduction reaction in the presence of surfactant is reported here for the first time.

## Introduction

Metallic nanomaterials with open curved surfaces having low-coordinated atomic sites, such as nanoporous/mesoporous structures,^1^ nanoframes,^2^ dendritic structures,^3^ and polyhedra/nanowires/nanorods,^4^ have attracted fast-growing attention in catalysis applications owing to their fascinating performance. In particular, metals with mesoporous structures possess a high surface area, three-dimensional molecular accessibility and can facilitate mass transfer from the outer surface to the inner structure.^5^ In addition, both inner and outer surfaces are composed of atoms with unsaturated valence, thus dramatically enhancing the electrocatalytic performance.^6^ Therefore, mesoporous metals offer a wide range of promising applications which cannot be satisfied by other silica-based mesoporous materials.

Currently, mesoporous metals have become a subject of extensive research as the presence of pores allows a fast and efficient transport of reactants.^7^ In order to precisely tune the properties of these materials, it is required to design their porous architecture. Several strategies, including dealloying techniques, galvanic replacement reactions, electrochemical depositions, and soft- and hard-templating methods, have been developed.^8^ Especially, soft-templating approach based on a simple chemical reduction has been considered as a more versatile, flexible, and effective strategy, which would be easily applicable to a large-scale production in the future. Until now, it should be noted that most of the studies reported that mesoporous metals limited to particles with irregular shape or to continuous films. The lack of control over the particle size and morphology is a serious problem, which needs to be overcome in order to further develop and implement mesoporous metals in electrocatalytic applications. Our target in this study is to design mesoporous Pt nanospheres (MPNs) with a narrow size distribution by a versatile chemical reduction approach.

To further enhance the catalytic performance of MPNs, here we focus on the design of “true” mesoporous structures as well as abundant active sites designed on the mesoporous surfaces. Although several solution phase approaches have been attempted to prepare Pt electrocatalysts, the obtained architectures are limited to only dendritic Pt nanoparticles possessing tiny and random interspaces (less than 5 nm).^9^ Mesoporous Pt nanoparticles/nanospheres are ideal to realize beneficial mass transport.^10^ Some effective additives have been utilized to create abundant catalytically active sites on the noble metal surfaces,^11^ because such additives are capable in tailoring the reaction kinetics of the precursors during the crystal growth. Therefore, in this study, we propose to prepare “true” mesoporous Pt nanospheres with abundant active sites on the porous surface by using a kinetically controlled chemical reduction, which has never been exploited in the synthesis of mesoporous metals. We strongly believe that it will be a promising strategy to produce highly performing electrocatalytic materials.

In this paper, we report the synthesis of MPNs with interconnected mesopores by a controlled chemical reduction in aqueous solution without the necessity of any seeds or organic solvents. In brief, MPNs are synthesized by reducing H_2_PtCl_6_ with ascorbic acid in the presence of a Pluronic F127 surfactant and KBr. It is found that F127 acts as a pore-directing agent, while the combination of bromide ions and dioxygen allows the porous structure to become interconnected and the surface to become catalytically active. Compared to commercial Pt black (PtB) and PtC-20% catalysts, our MPNs exhibit an enhanced electrocatalytic activity and structural thermostability.

## Experimental section

### Preparation of MPNs

In a typical synthesis of mesoporous Pt nanospheres (MPNs), 200 mg KBr and 30 mg Pluronic F127 were dissolved in 2 mL deionized water. After complete dissolution, 0.75 mL H_2_PtCl_6_ solution (40 mM) and 2 mL ascorbic acid (AA) solution (0.1 M) were added. The mixed solution was then kept in a water bath for 12 h at 70 °C. Finally, the sample was collected by centrifugation at 14 000 rpm for 20 min and the residual Pluronic F127 was removed by three consecutive washing/centrifugation cycles with ethanol and water.

### Electrochemical measurements of methanol oxidation reaction (MOR)

Cyclic voltammograms (CV) and chronoamperometric (CA) experiments were performed using a CHI 842B electrochemical analyzer (CHI Instruments, USA). A conventional three-electrode cell was used, including an Ag/AgCl (saturated KCl) reference electrode, a platinum wire as a counter electrode, and a modified glassy carbon electrode (GCE, 3 mm in diameter) as a working electrode. The modified GCE was coated with the sample (5.0 μg) and dried at room temperature. Then, 5.0 μL of Nafion (0.05 wt%) was coated on the surface of the modified GCE and dried before electrochemical experiments. Prior to the electrochemical measurements, the GCE modified with the as-prepared sample was electrochemically activated by a potential cycling between –0.2 and +1.5 V (*vs.* Ag/AgCl) in 0.5 M H_2_SO_4_ until the obtained CVs became characteristic of a clean Pt electrode. Methanol electro-oxidation measurements were performed in a solution of 0.5 M H_2_SO_4_ containing 0.5 M methanol at a scan rate of 50 mV s^–1^. The chronoamperometric measurement was carried out at a constant potential of 0.6 V for 3000 s.

### Electrochemical measurements of oxygen reduction reaction (ORR)

CV measurements were obtained from a similar setup to the MOR measurement. A conventional three-electrode cell was used, including an Ag/AgCl (saturated KCl) reference electrode, a platinum wire as a counter electrode, and a modified rotating ring-disk electrode (RRDE, 4 mm in diameter) as a working electrode. The working electrode was prepared as follows. 2 mg of catalyst was dispersed in a mixture of water (0.4 mL) and Nafion (5 wt%, 10 μL) under ultrasonication for 30 min to obtain a uniform suspension of the catalyst. Then, 5 μL of the above suspension was dropped on the disk electrode surface of the RRDE and dried at room temperature. The electrochemical measurements were performed in 0.1 M HClO_4_ saturated with O_2_ at a potential range between –0.2 V and 1.0 V (*vs.* Ag/AgCl) with a scan rate of 10 mV s^–1^ and a typical rotating speed of 1600 rpm. In this work, electrochemical tests for different electrocatalysts were repeated three times.

The rotating ring-disk electrode (RRDE) was used to study the reduction pathway of oxygen. The ring electrode current, which corresponds to the oxidation of intermediate (hydrogen peroxide), was recorded together with the disk electrode current. The electron number (*n*) transferred per oxygen molecule involved in the ORR was calculated from RRDE voltammograms according to the following equation:*n* = 4*I*_d_/((*I*_d_ + *I*_r_)/*N*)where *I*_d_ is the disk current, *I*_r_ is the ring current and *N* = 0.4 is the current collection efficiency of the Pt ring electrode.

### Characterization

Scanning electron microscopy was carried out using a Hitachi SU-8000 microscope operated at 5 kV. Transmission electron microscopy and high-angle annular dark-field scanning TEM (HAADF-STEM) observations were performed using a JEOL JEM-2100F operated at 200 kV equipped with an energy-dispersive spectrometry analyzer. The samples for TEM characterizations were prepared by depositing a drop of the diluted colloidal suspension on a grid. Wide-angle powder X-ray diffraction (XRD) patterns were acquired with a Rigaku Rint 2500 diffractometer with monochromated Cu-Kα radiation. Low-angle XRD patterns were obtained by using a NANO VIEWER (Rigaku, Japan) equipped with a Micro Max-007 HF high-intensity micro-focus rotating anode X-ray generator. X-Ray photoelectronic spectroscopy (XPS) spectra were obtained at room temperature by using a JPS-9010TR (JEOL) instrument with an Mg-Kα X-ray source. All the binding energies were calibrated *via* referencing to C 1s binding energy (284.6 eV).

## Results and discussion

**Fig. 1:**
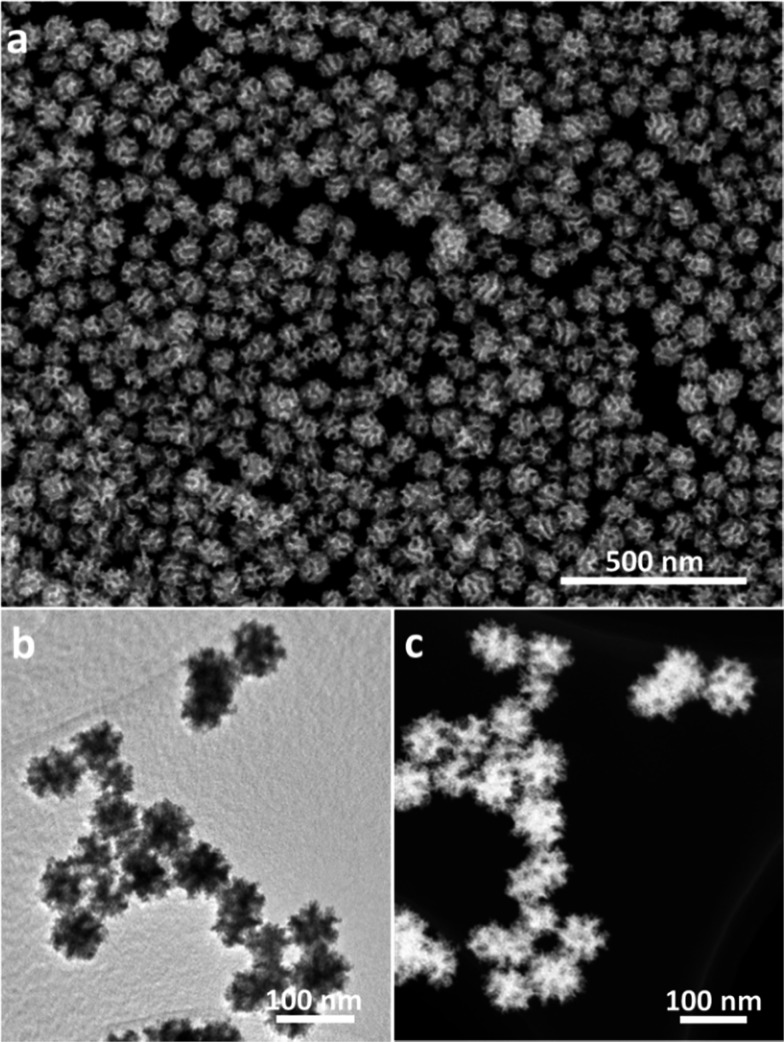
(a) Low-magnification SEM, (b) TEM and (c) HAADF-STEM images of the MPNs prepared under the typical condition.

**Fig. 2:**
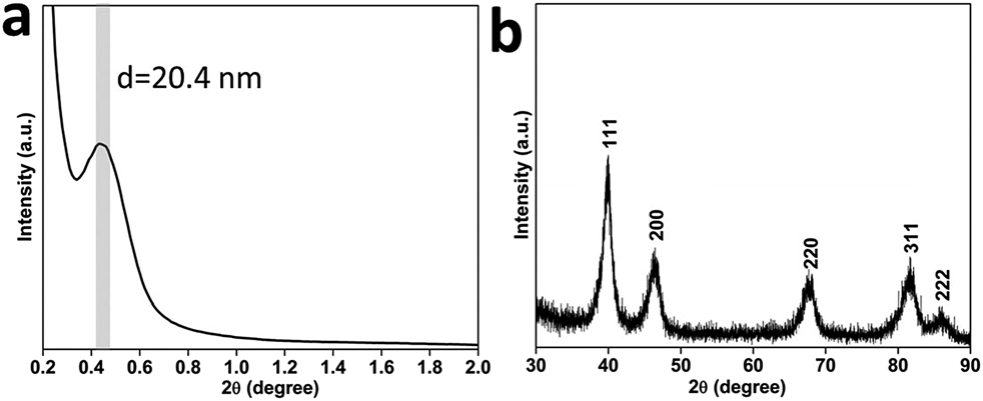
(a) Low- and (b) wide-angle XRD patterns of MPNs.

Scanning electron microscope (SEM) and transmission electron microscopy (TEM) were employed to characterize the structures of the product. The nanospheres are well-dispersed and highly uniform in shape and size (Fig. 1a and S1†). An average size of around 70 nm is determined from statistical measurements over 200 nanospheres (Fig. S2†) and the pores on the surface have a size distribution centered on 11 ± 1 nm. The porous structure and size are distinctly different from the dendritic nanoparticles prepared previously.^9^ The different contrasts observed on the TEM image evidence that the mesopores are deeply embedded and interconnected in the nanospheres (Fig. 1b). These mesoporous structures are further presented in a high-angle annular dark-field scanning TEM (HAADF-STEM) image (Fig. 1c). Considering the average pore diameter and wall thickness measured from the SEM analysis to be 11 and 9–10 nm, respectively, the average pore-to-pore distance is estimated to be roughly 20–21 nm. This is in good agreement with the low-angle X-ray diffraction (XRD) spectrum displaying a sharp peak at 0.43° (*d* = 20.3 nm) (Fig. 2a), which is also a strong evidence of the high uniformity of the sample.

The wide-angle XRD pattern of the as-prepared MPNs can be assigned to a pure face-centered cubic (*fcc*) Pt structure (Fig. 2b) and energy dispersive X-ray spectroscopy measurements further confirmed that the sample is free of other elemental impurities (Fig. S3†). X-Ray photoelectron spectroscopy (XPS) was then employed to analyze the valence state of the surface Pt in the MPNs. For comparison, commercially available Pt black (PtB) was similarly characterized. It can be clearly noticed in Fig. S4† that both samples show doublet peaks assignable to Pt^0^ 4f_7/2_ and Pt^0^ 4f_5/2_, respectively, indicating the existence of metallic state Pt.^12^ Considering the resolution depth of Pt photoelectron spectra at the conditions (*i.e.*, 1.5 nm) of an inelastic-mean-free-path,^13^ the spectra are considered to be composed of the contribution from the top 2–3 surface atomic layers.

**Fig. 3:**
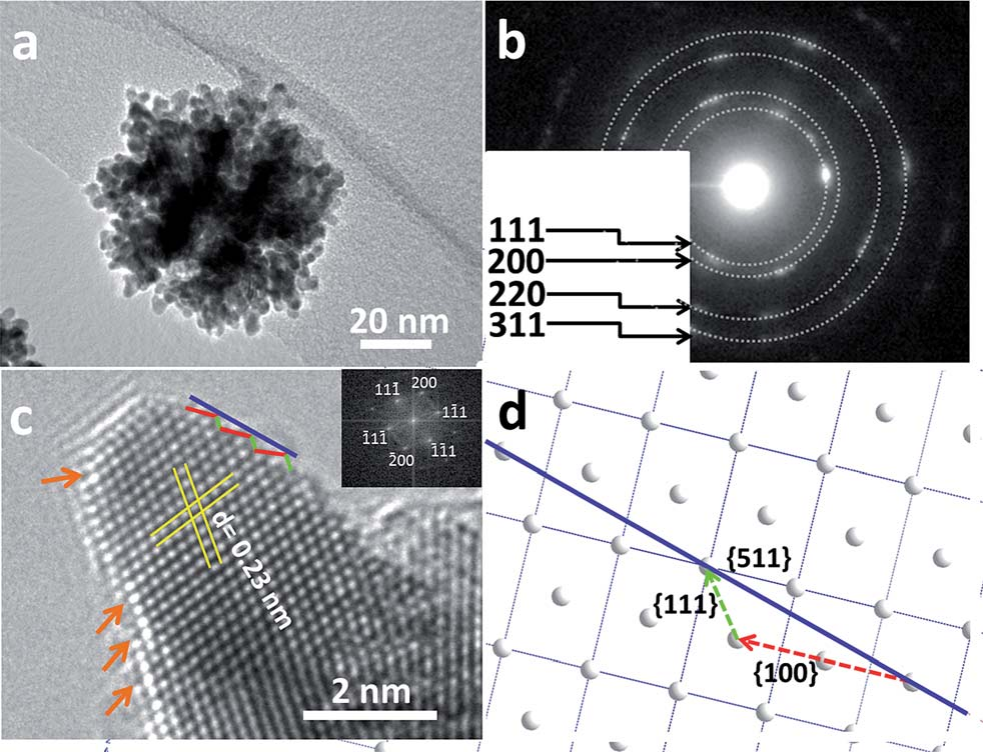
(a) Typical TEM image and (b) the corresponding selected area ED pattern of a single MPN. (c) HRTEM image focused on the edge of Pt pore walls projected along the 011 direction. (d) Schematic illustration for the exposed high-index facet. The inset image in panel (c) shows the corresponding FFT patterns of the HRTEM image.

In order to obtain more information on the surface atomic structure, high-resolution TEM (HRTEM) and TEM images of the MPNs were acquired. It can be observed that an entire nanosphere is typically composed of interconnected nanocrystals sizing from 6 to 8 nm (Fig. 3a). The mesoporous structure can be clearly distinguished because of differences in the contrast. The selected-area electron diffraction (ED) pattern from Fig. 3b along with the clear lattice fringes observed in the HRTEM image (Fig. 3c) evidence the high degree of crystallization of the pore walls. Also, the lattice spacing of 0.23 nm is in good agreement with the *fcc* Pt {111} crystal plane. The surface of the branched Pt nanocrystals is enclosed by {111} and {100} facets along with a large number of atomic steps, as indicated by the arrows in Fig. 3c. Furthermore, some high-index facets (*e.g.*, {511}) were confirmed from the edge surface. A scheme shown in Fig. 3d illustrates the positioning model for the high-index facet. The favorable molecule adsorption attributed to unsaturated atomic structures has been proved to be more active than highly coordinated atomic surfaces.^14^

To better understand the formation mechanism of the MPNs, the roles of the reagents were investigated in detail. It has previously been reported that nonionic surfactants, such as F127 and P123, can serve as structure-directing agents in the preparation of mesoporous oxides and carbons.^15^ As shown in Fig. S5a,† in the absence of F127, only micrometer-sized Pt spheres with non-porous structure are obtained. When a small amount of F127 (2.0 mg) is added, monodispersed Pt nanoparticles with shallow pores start to appear (Fig. S5b†). Further increasing the amount of F127 (up to 10 mg) leads to the formation of concave mesopores on the surface of the Pt nanoparticles (Fig. S5c†). With further increasing the amount of F127 (up to 30 mg), the desired mesoporous Pt nanospheres can be obtained (Fig. S5d†).

The formation of the mesoporous particles is completely different from our previous works, where porous or dendritic Pt nanoparticles with narrow interspaces of less than 5 nm were obtained through fast reduction reactions.^8a,9a^ In this work, we believe that the slow deposition rate is responsible for the retention of the micelle arrangement at the greatest extent and thus, for the formation of well-defined mesoporous structures.^16^ Although the reduction kinetics can often be controlled by varying the reduction power of the reducing agent, here, we utilize another approach, *i.e.*, the ligand exchange of Pt precursor by adding bromide ions and oxygen to slow down the reduction reaction.

The mechanism of these two additives can be accounted for by the following. The presence of bromide ions shifts the redox potential of the Pt ions toward a more negative potential through ligand exchange reactions to form [PtBr_6_]^2–^ with a redox potential more negative (0.61 V *vs.* SHE) than that of [PtCl_6_]^2–^ (0.73 V *vs.* SHE), namely, the reduction of [PtBr_6_]^2–^ is slower. This ligand exchange reaction was confirmed by UV-vis measurements (Fig. S6†). The presence of oxygen shifts the reduction potential toward a more positive potential.

The roles of Br^–^ and O_2_ in the formation of the Pt nanospheres were carefully studied by a series of control experiments: samples were synthesized in the presence of oxygen but without bromide ions (Fig. S7a†), and in the presence of bromide ions but without oxygen (Fig. S7b†). In both cases, the reaction yields disconnected cavities on the surface of the Pt nanoparticles possessing a broad size distribution. The structures with interconnected mesopores can only be obtained when both Br^–^ and O_2_ are simultaneously involved in the reaction (Fig. 1a and S1†). The slow reaction favors a thermodynamic growth, which induces the exposure of {111} facets. Also, the Br^–^ ions are known to selectively adsorb on and preferably expose Pt {100} facets.^17^ Therefore, in this study, the designed pore surfaces consisting of the interlaced Pt {111} and {100} facets (Fig. 3c) is proved to enhance the oxygen reduction reaction activity, which is probably ascribed to a synergetic effect between different facets.^18^ To the best of our knowledge, such mesoporous Pt nanospheres with designed pore surface were firstly reported.

Thus, we believe that this slow metal growth is beneficial for the formation of uniformly sized mesopores and particles as it provides enough time for the surfactants to adhere to the metal seeds and effectively act as a template for the subsequent growth (Fig. S8†). When ascorbic acid is substituted with stronger reducing agents (*e.g.*, hydrazine), the reduction rate increases significantly, leading to non-porous and inhomogeneous nanoparticles (Fig. S9†).

**Fig. 4:**
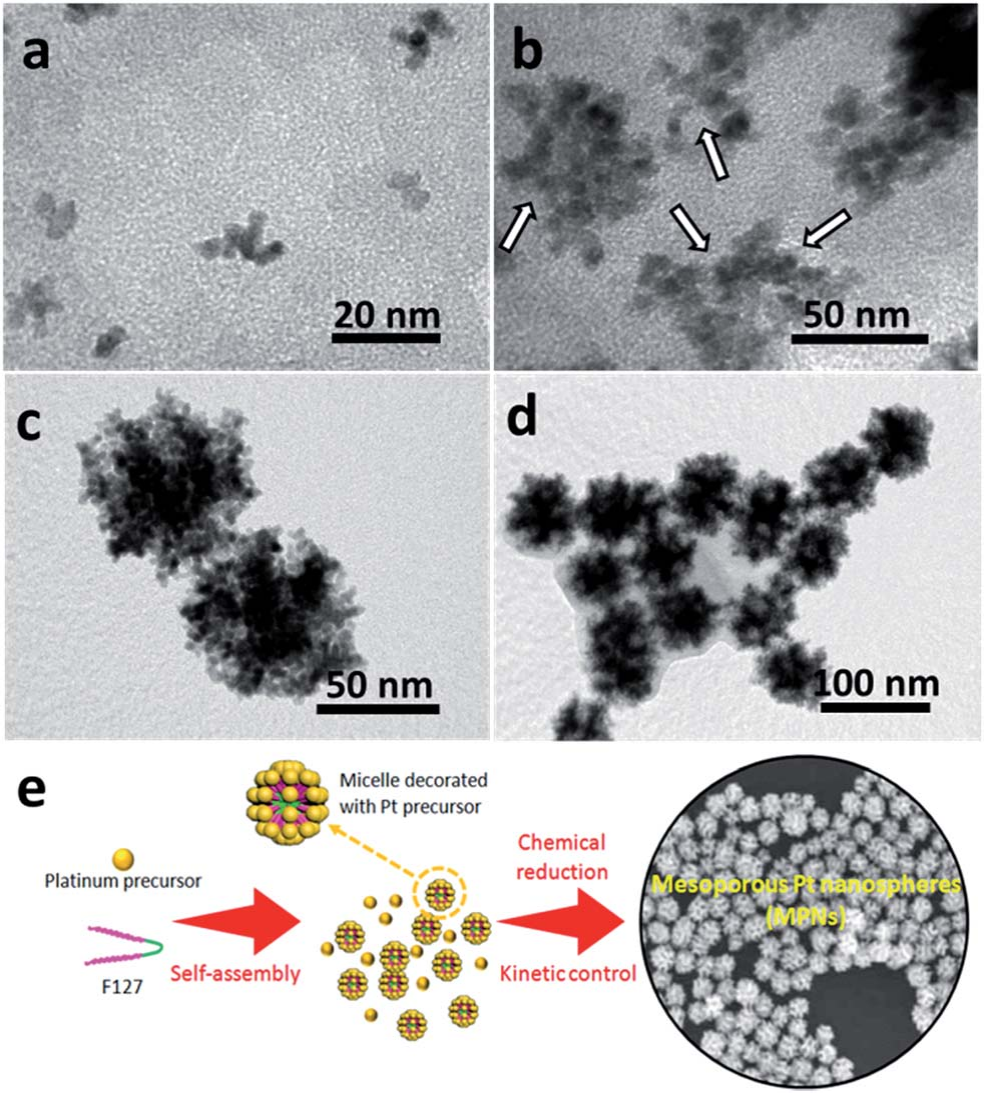
TEM images of Pt samples taken after different reaction periods: (a) 3 h, (b) 4 h, (c) 6 h and (d) 12 h, respectively. (e) Schematic illustration for the synthesis of MPNs.

The intermediate products at different reaction times were also investigated through TEM observation of the samples taken from the reaction flask after various periods of time (Fig. 4). Within 3 h, almost no precipitate can be observed, indicating that the reduction rate is relatively slow. The little amount of particles collected formed small non-uniform aggregates (Fig. 4a). With increasing the reaction time (4 h), the particle size increases by the continuous growth of fine particles (Fig. 4b). It is noted that, at this stage, concave or porous structures start to be formed on the edges of the particles, as indicated by the arrows. After 6 h, the MPNs are formed (Fig. 4c) and the morphology of the nanostructure remains stable even after longer reaction time (up to 12 h, Fig. 4d). On the basis of these results, the mechanism responsible for the formation of MPNs can be described as follows. At the initial stage, F127 serves as a protecting agent and stabilizes the primary Pt clusters through the interactions with the ethylene oxide (EO) chains of the F127 micelles.^19^ As the reaction proceeds, the reduced Pt clusters with preferentially exposed {100} facets from the solution continue to deposit on the spherical F127 micelles. Because of the extremely slow reduction rate caused by the ligand binding, there is enough time for F127 to effectively act as a template and to form an open mesoporous structure. The synthesis process of MPNs is schematically illustrated in Fig. 4e.

To investigate the nature of the Pt surface obtained through the slow reduction reactions in the presence of bromide ions, the surface crystallography of the MNPs was investigated by cyclic voltammetry and compared with those of dendritic Pt nanoparticles with shallow concave pores (DPNs) prepared in the presence of oxygen but without adding KBr (Fig. S7a†), and also with PtC-20% and PtB catalysts. The cyclic voltammograms (CVs) of MPNs and DPNs exhibit the typical characteristics of hydrogen and oxygen adsorption/desorption on polycrystalline Pt surface (Fig. S10†), while the CVs of the PtC-20% and PtB samples do not show any sharp peaks in the hydrogen region (*i.e.*, below 0.2 V *vs.* Ag/AgCl), which are similar to that of Pt {111} surface.^20^ MPNs and DPNs have a hydrogen desorption peak around –0.08 V *vs.* Ag/AgCl, which is assignable to {110} surfaces, and another sharp peak around 0.08 V, which can be assigned to a few atoms wide {100} surface.^21^ Interestingly, we found that only MPNs have an extra third peak between the peaks previously mentioned, which can be assigned to low coordination Pt sites.^21,22^ These clearly show that our nanostructured samples have more surface step defects than typical Pt nanoparticles.

**Fig. 5:**
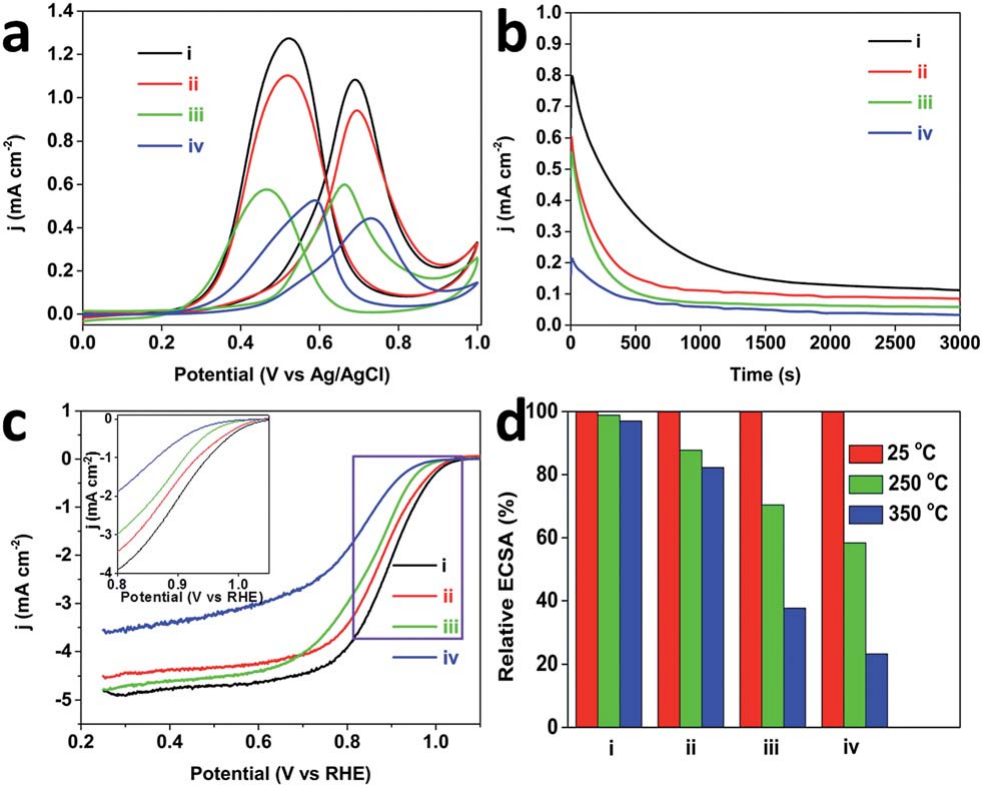
(a) Cyclic voltammograms of MOR recorded in 0.5 M H_2_SO_4_ + 0.5 M CH_3_OH at a scan rate of 50 mV s^–1^, (b) chronoamperometric curves (recorded at 0.6 V) obtained in 0.5 M H_2_ SO_4_ + 0.5 M CH_3_OH, (c) ORR polarization curves recorded in O_2_-saturated 0.1 M HClO_4_ solution with a sweep rate of 10 mV s^–1^ and a rotation rate of 1600 rpm, and (d) ECSA retention after thermal treatment at various temperatures for 3 h [(i) MPNs with interconnected mesopores, (ii) DPNs, (iii) PtC-20% catalyst, and (iv) PtB catalyst, respectively]. The inset image in panel (c) shows enlargement of the rectangle area. The currents in panel (a) are normalized by the ECSA, whereas, the currents in panel (c) are normalized by the electrode geometric surface area.

Inspired by these attractive properties (*e.g.*, low-coordinated atomic surfaces), both the methanol oxidation reaction (MOR) and oxygen reduction reaction (ORR) were performed to evaluate the electrochemical performance of the MPNs. Fig. 5a shows typical CV curves of MOR catalyzed with the MPNs, DPNs, PtC-20% and PtB catalysts. The current densities were normalized by the Pt electrochemical surface area (ECSA) (MPNs (25.1 m^2^ g^–1^ Pt), DPNs (17.1 m^2^ g^–1^ Pt), PtC-20% (54.0 m^2^ g^–1^ Pt) and PtB (14.3 m^2^ g^–1^ Pt)). The peak current density of the MPNs (1.1 mA cm^–2^) is much higher than that of the DPNs (0.95 mA cm^–2^), PtC-20% (0.61 mA cm^–2^) and PtB (0.46 mA cm^–2^). The reason for this superior activity arises from a large number of low-coordinated atoms present on the Pt surfaces, which facilitate the breaking of C–H bonds during the decomposition of methanol.^23^ The chronoamperometric measurement at 0.6 V over 3000 s proved that the MPNs possess a better stability during the electrochemical measurement, in comparison to the other catalysts (Fig. 5b). This suggests that the mesoporous structure of the MPNs can be well retained even after the long-term stability measurement (Fig. S11†).

**Fig. 6:**
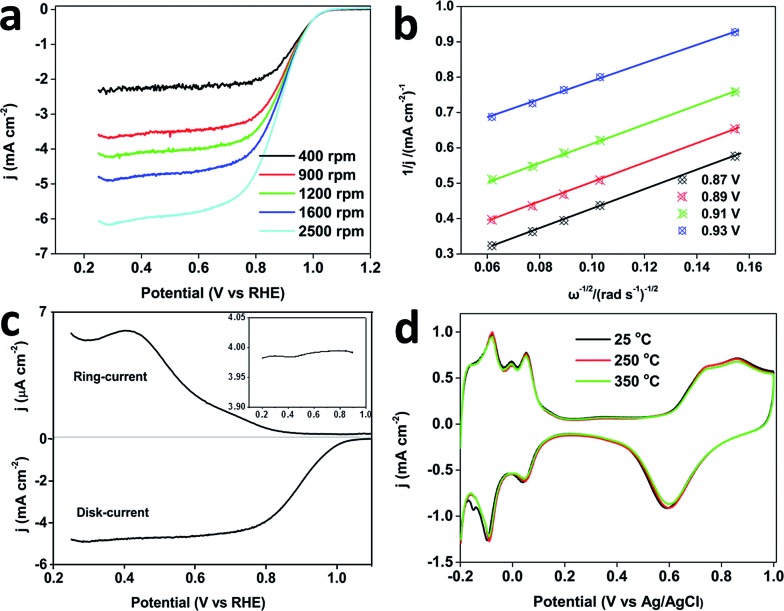
(a) ORR polarization curves of MPNs at different rotation rates, and (b) corresponding Koutecky–Levich (K–L) plots of the MPNs at different potentials. (c) The current collected on disk and ring electrodes catalyzed by MPNs. Inset shows the electron number transferred in ORR calculated by using the current collected on the rotating ring-disk electrode. (d) Cyclic voltammograms of the MPNs before (black plot) and after thermal treatment at 250 °C (red plot), or 350 °C (green plot) for 3 h, which are recorded in 0.5 M H_2_SO_4_ at a sweep rate of 50 mV s^–1^. All the ORR measurements were carried out in O_2_-saturated 0.1 M HClO_4_ solution. The K–L plots show the inverse current density (*j* ^–1^) as a function of the inverse of the square root of rotation speed (*ω* ^–1/2^) at various potentials. All the currents are normalized by the electrode geometric surface area.

The ORR was further studied in O_2_ saturated 0.1 M HClO_4_. The MPNs show a more positive half-wave potential (0.88 V) than that of PtB (0.78 V), DPNs (0.86 V) and PtC-20% (0.84 V) (Fig. 5c). The onset potential of the MPNs is positively shifted compared to the other samples. To further study the kinetics of the ORR, the polarization curves of the MPNs-modified rotation ring disk electrode (RRDE) were recorded at different rotation speeds. The limiting current density increases with increasing the rotation speed (Fig. 6a). The corresponding Koutecky–Levich (K–L) plots at different potential values are plotted and the number of electrons transferred was calculated by the K–L equation,^24^ which is expressed by:

1 *j*^–1^ = *j*_K_^–1^ + *j*_L_^–1^ = *j*_K_^–1^ + *B*^–1^*ω*^–1/2^

2 *B* = 0.62*nFC*_0_*D*_0_^2/3^*ν*^–1/6^

where *j* is the measured current density; *j*_K_ and *j*_L_ are the kinetic- and diffusion-limited current densities; *ω* is the angular frequency of rotation; *n* represents the overall number of electrons transferred in oxygen reduction; *F* is the Faraday constant (*F* = 96 485 C mol^–1^); *C*_0_ is the bulk concentration of O_2_ in 0.1 M HClO_4_ (1.26 × 10^–6^ mol cm^–3^); *D*_0_ is the diffusion coefficient of O_2_ in 0.1 M HClO_4_ solution (1.93 × 10^–5^ cm^2^ s^–1^); *ν* is the kinematic viscosity of the electrolyte (1.01 × 10^–2^ cm^2^ s^–1^). The number of electrons transferred (*n*) can be obtained from the slope of the K–L plots.

Fig. 6b shows four linear K–L plots at different potentials, suggesting the first-order reaction kinetics of oxygen reduction catalysed by the MPNs from 0.825 to 0.925 V. The *n* value calculated from the equation: *n* = 4*I*_d_/((*I*_d_ + *I*_r_)/*N*)^25^ (where *I*_d_ is the disk current, *I*_r_ is the ring current, and *N* = 0.4 is the current collection efficiency of Pt ring electrode), is in the range of 3.7–4.0, which is in good consistency with the number of electrons derived from the RRDE voltammograms (Fig. 6c). All these results evidence that the ORR catalyzed by the MPNs follows a four-electron pathway.^26^

It is well known that structural thermostability is a very important factor for practical catalysts because the performance highly depends on their ability to retain their shape and structure. In order to investigate the structural thermostability, the MPNs, DPNs, PtC-20% and PtB were treated at 250 or 350 °C for 3 h. After calcination, the CV plots were recorded in 0.5 M H_2_SO_4_ at a scan rate of 50 mV s^–1^ and the Pt ECSA of each sample was calculated and compared. Fig. 5d shows that 97% of initial ECSA of the MPNs can be retained even after the thermal treatment at 350 °C, which is much higher than for the DPNs (82%), PtC-20% (38%) or PtB (23%), showcasing the MPNs for their excellent thermostability. Because the well-defined mesoporous structure was less vulnerable to particle aggregation, the MPNs retain most of their ECSA after the treatment at 350 °C, in great contrast to the low thermal stabilities of the DPNs caused by structural shrinkage.^9^ The serious decrease of ECSAs for PtC-20% and PtB can be attributed to the aggregation of the nanoparticles and the collapse of the carbon support at higher temperatures (Fig. S12†). The morphologies of MPNs do not show any changes either (Fig. S13†). Also, the CV curves are perfectly maintained even after thermal treatment at 250 and 350 °C (Fig. 6d).

## Conclusions

We have successfully synthesized mono-dispersed MPNs with interconnected mesopores by a one-pot method. Through systematic studies, we found that the kinetically controlled reduction reactions in the presence of F127 structure-directing agent are responsible for the formation of active interconnected mesopores in the Pt nanoparticles. Interestingly, our MPNs not only show an enhanced electrocatalytic performance but also exhibit a high structure thermostability, which make them a promising catalyst for practical applications. We strongly believe that this work will provide new insights towards the creation of novel metal-based materials.
