# Supplementary material for: Mesoporous Pt nanospheres with designed pore surface as highly active electrocatalyst
Source: Chem Sci. 2015 Dec 8;7(2):1575–81. doi: 10.1039/c5sc03779d (PMC5530946; doi:10.1039/c5sc03779d)
Supplement: Supplementary file 1 [file SC-007-C5SC03779D-s001.pdf]

This journal is © The Royal Society of Chemistry 2015

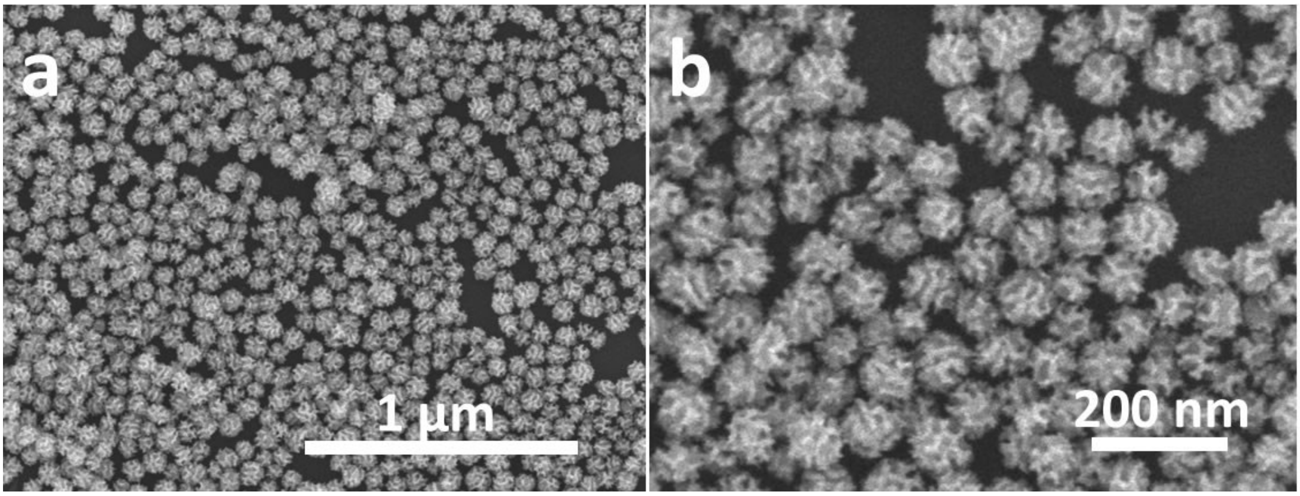


**Figure S1 |** Typical SEM images of the MPNs at low and high magnifications.

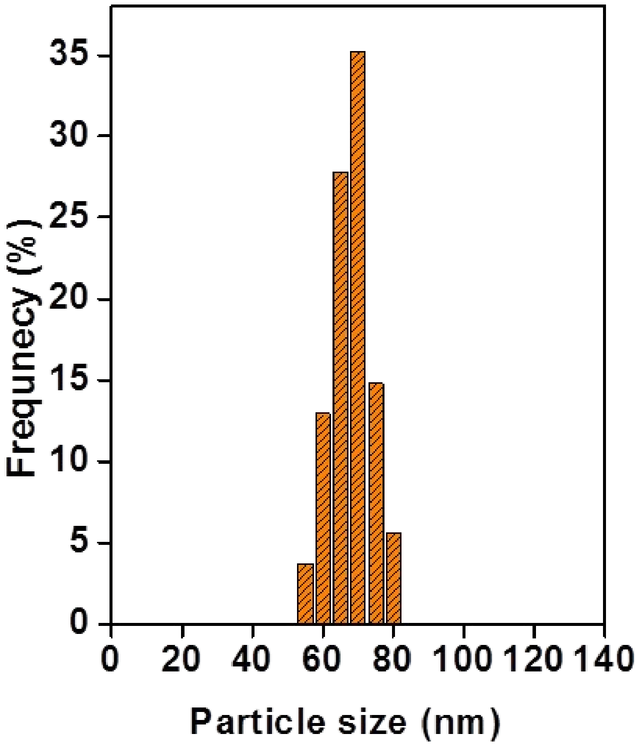


**Figure S2 |** Histogram of particle size distribution of the MPNs with interconnected mesopores.

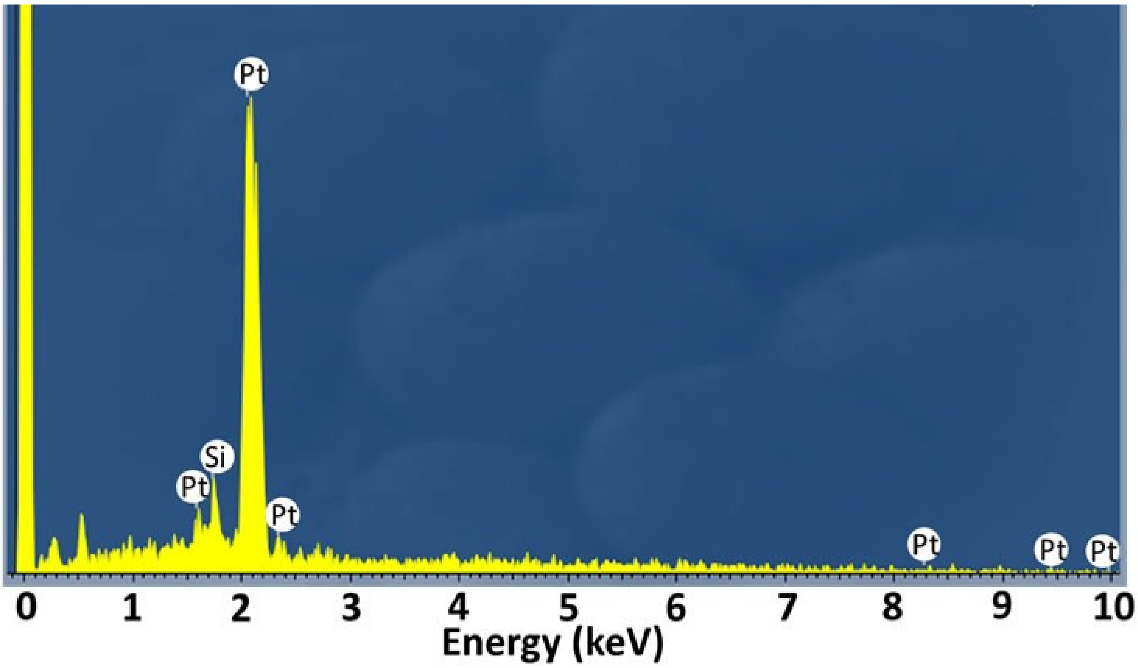


**Figure S3 |** A representative EDX spectrum of MPNs. In the EDX spectrum, the additional signal originates from the Si substrate.

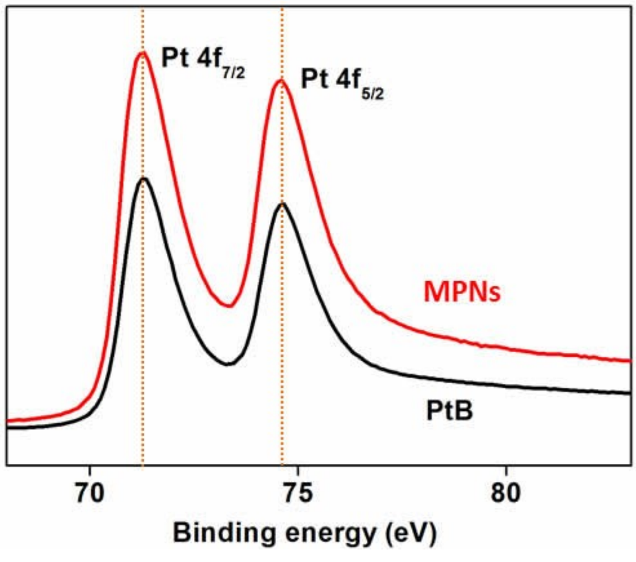


**Figure S4 |** Pt 4*f* XPS data of MPNs and PtB.

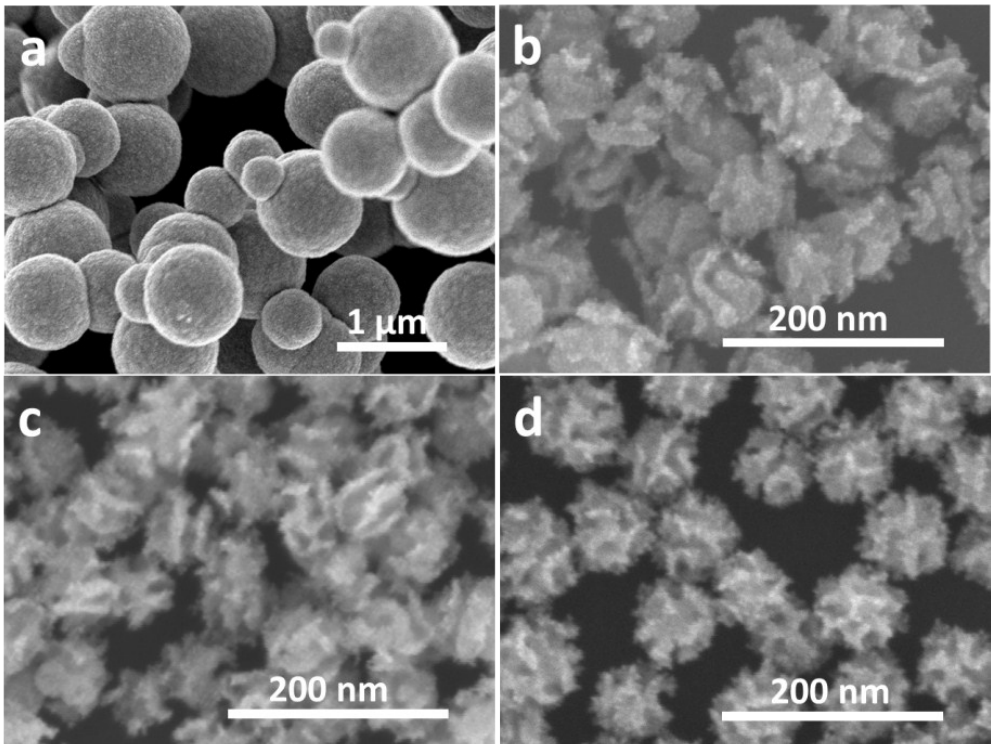


**Figure S5 |** SEM images of the samples prepared under typical conditions with different amounts of F127 [(a) 0 mg, (b) 2.0 mg, (c) 10 mg, and (d) 30 mg, respectively].

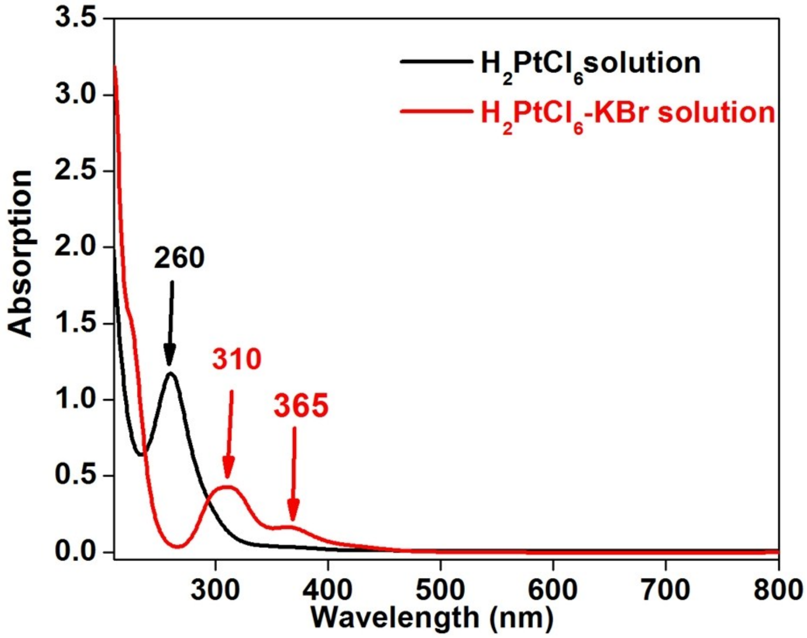


**Figure S6 |** UV-vis spectrum of (black plot) $H_2PtCl_6$ solution only and (red) $H_2PtCl_6$ solution after addition of KBr.

**Comment in Figure S6:** The absorption peak located at 260 nm in the $[PtCl_6]^{2-}$ solution corresponds to the absorption of $[PtCl_6]^{2-}$ complexes.[R1] After addition of KBr, the absorption peak at 260 nm disappears and two new characteristic features located at 310 and 365 nm can be observed, indicating that $[PtCl_6]^{2-}$ was successfully replaced by $[PtBr_6]^{2-}$ complexes.[R2]

**Reference:**


[R1] a) Tang, Z.; Geng, D.; Lu, G. *J. Colloid Interface Sci*. **2005**, 287, 159-166; b) Hikosaka, K.; Kim, J.; Kajita, M.; Kanayama, A.; Miyamoto, Y. *Colloid Surf. B* **2008**, 66, 195-200.

[R2] a) Dabestani, R.; Wang, X.; Bard, A.; Campon, A.; Fox, M. A.; Webber, S.; White, J. M.; *J. Phys. Chem.* **1986**, 90, 2729-2732; b) Glebov, E. M.; Plyusnin, V. F.; Grivin, V.P.; Venediktov, A. B.; Korenev, S. V.; *Russ. Chem. B. Int. Ed.* **2007**, 56, 2357-2363.

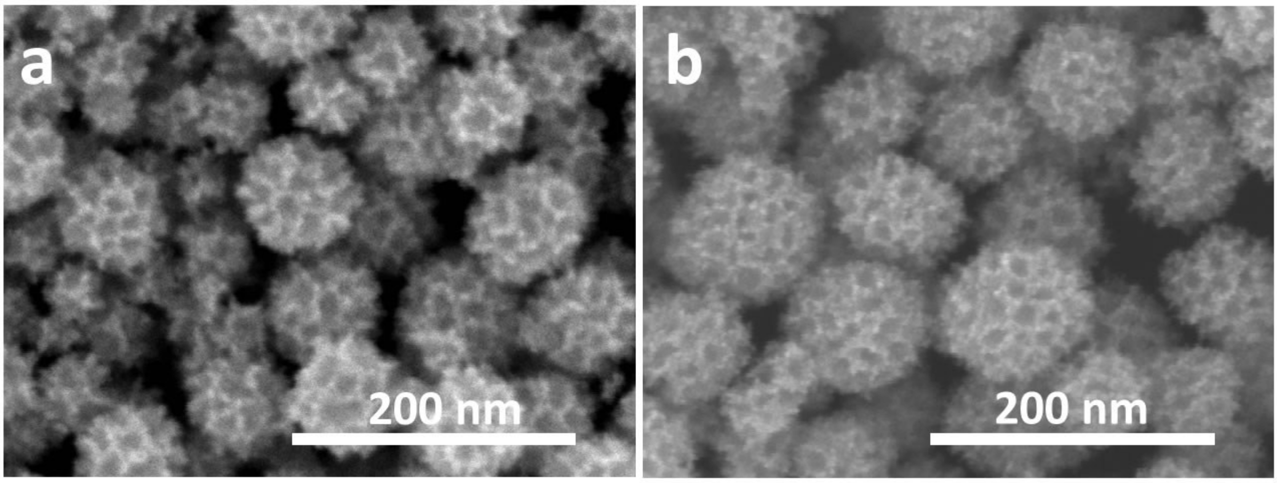


**Figure S7 |** SEM images of samples prepared (a) with oxygen but without KBr and (b) with KBr but without oxygen.

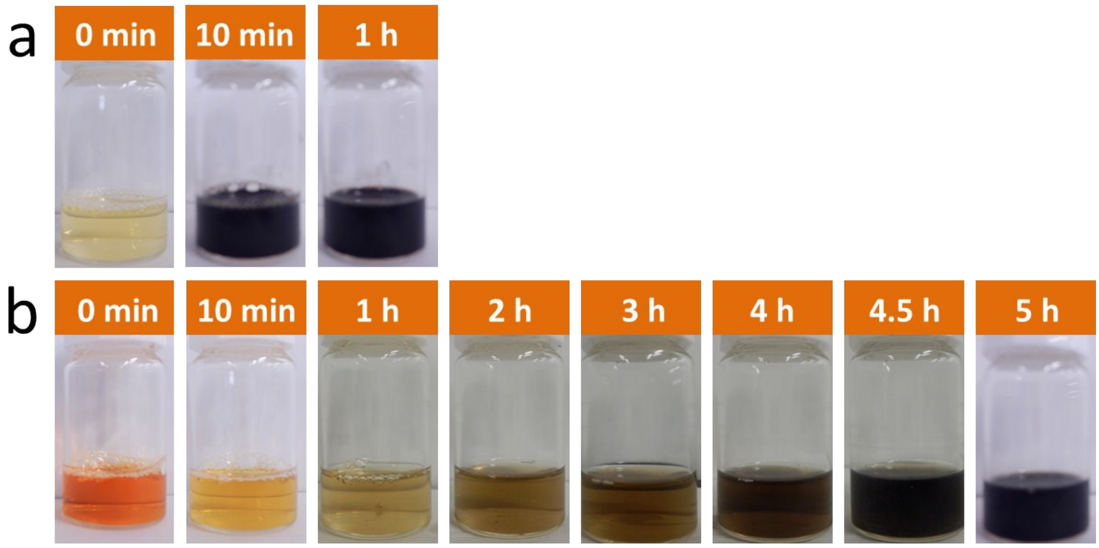


**Figure S8 |** Photographs of colloidal suspensions of (a) $H_2PtCl_6$ solution, and (b) $H_2PtCl_6$-KBr solution taken at different reaction times at 70 $^{o}$C.

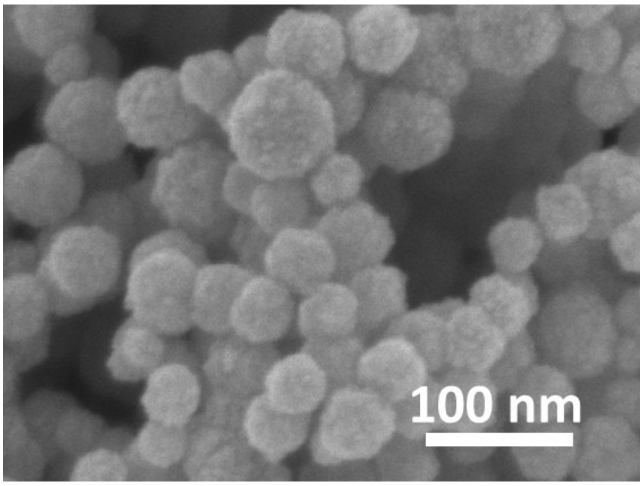


**Figure S9 |** SEM image of the as-prepared Pt sample prepared by substituting ascorbic acid with hydrazine.

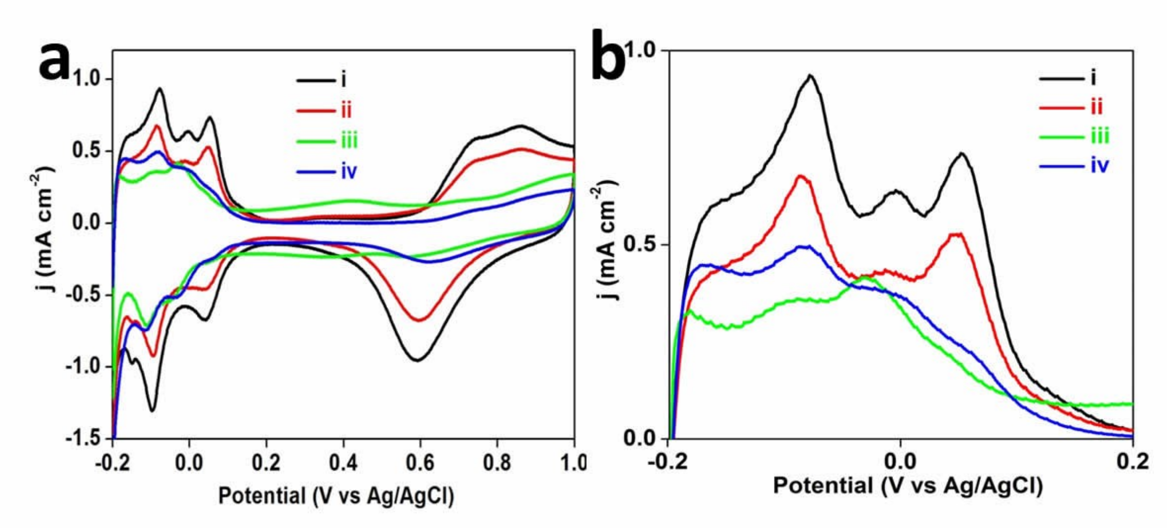


**Figure S10** | Cyclic voltammograms of (i) MPNs, (ii) DPNs, (iii) PtC-20%, and (iv) PtB catalysts recorded in 0.5 M $H_2SO_4$ solution at a scan rate of 50 mV $s^{-1}$. All the currents are normalized by electrode geometric surface area.

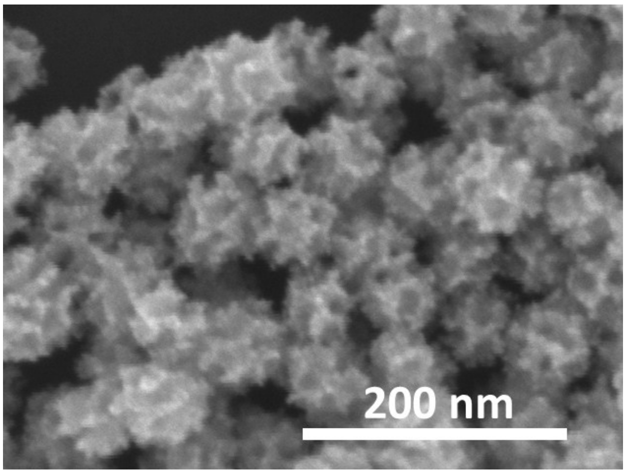


**Figure S11 |** SEM images of MPNs after electrocatalytic measurements.

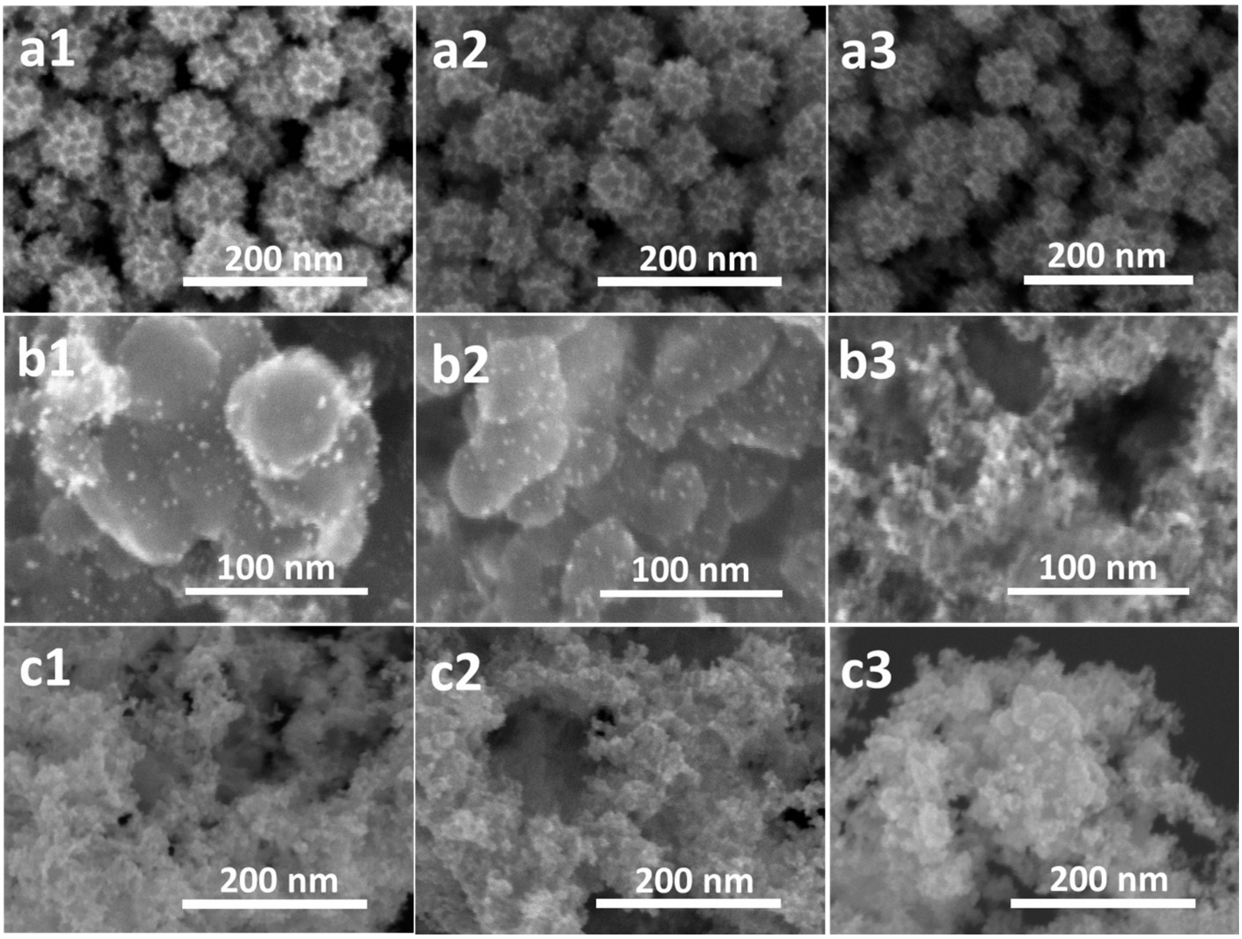


**Figure S12 |** SEM image of (a) DPNs, (b) 20%-PtC, and (c) PtB catalysts after thermal treatment at different temperatures [(a1, b1, c1) 25 $^oC$, (a2, b2, c2) 250 $^oC$, and (a3, b3, c3) 350 $^oC$, respectively].

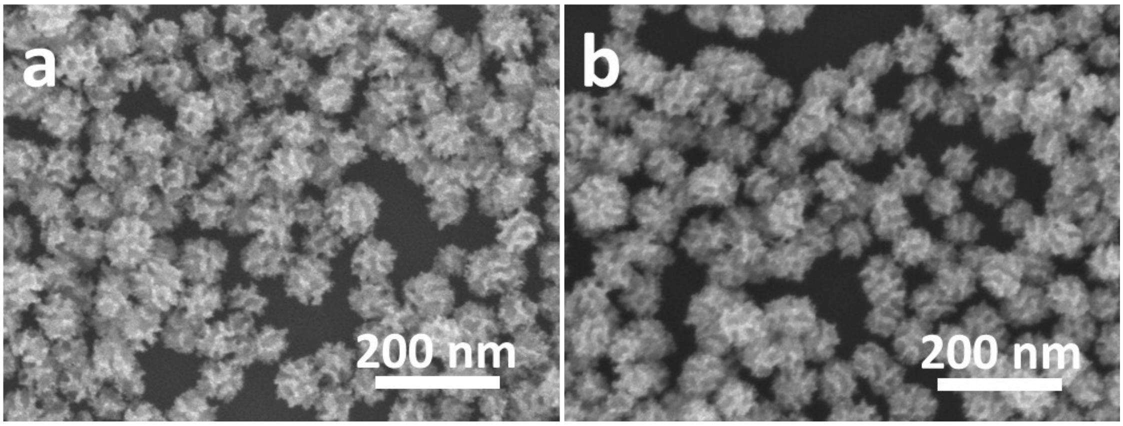


**Figure S13 |** SEM images of the MPNs after thermal treatment at (a) 250 $^{o}$C and (b) 350 $^{o}$C for 3 hours.
